# Inkjet-Printed and Electroplated 3D Electrodes for Recording Extracellular Signals in Cell Culture

**DOI:** 10.3390/s21123981

**Published:** 2021-06-09

**Authors:** Leroy Grob, Philipp Rinklin, Sabine Zips, Dirk Mayer, Sabrina Weidlich, Korkut Terkan, Lennart J. K. Weiß, Nouran Adly, Andreas Offenhäusser, Bernhard Wolfrum

**Affiliations:** 1 Neuroelectronics, Department of Electrical and Computer Engineering, MSB, MSRM, Technical University of Munich, Boltzmannstraße 11, 85748 Garching, Germany; leroy.grob@tum.de (L.G.); philipp.rinklin@tum.de (P.R.); sabine.zips@tum.de (S.Z.); k.terkan@tum.de (K.T.); lennart.weiss@tum.de (L.J.K.W.); nouran.adly@tum.de (N.A.); 2 Institute of Biological Information Processing (IBI-3), Forschungszentrum Jülich GmbH, 52425 Jülich, Germany; dirk.mayer@fz-juelich.de (D.M.); s.weidlich@fz-juelich.de (S.W.); a.offenhaeusser@fz-juelich.de (A.O.)

**Keywords:** 3D electrodes, inkjet printing, electrodeposition, impedance spectroscopy, cyclic voltammetry, bioelectronics

## Abstract

Recent investigations into cardiac or nervous tissues call for systems that are able to electrically record in 3D as opposed to 2D. Typically, challenging microfabrication steps are required to produce 3D microelectrode arrays capable of recording at the desired position within the tissue of interest. As an alternative, additive manufacturing is becoming a versatile platform for rapidly prototyping novel sensors with flexible geometric design. In this work, 3D MEAs for cell-culture applications were fabricated using a piezoelectric inkjet printer. The aspect ratio and height of the printed 3D electrodes were user-defined by adjusting the number of deposited droplets of silver nanoparticle ink along with a continuous printing method and an appropriate drop-to-drop delay. The Ag 3D MEAs were later electroplated with Au and Pt in order to reduce leakage of potentially cytotoxic silver ions into the cellular medium. The functionality of the array was confirmed using impedance spectroscopy, cyclic voltammetry, and recordings of extracellular potentials from cardiomyocyte-like HL-1 cells.

## 1. Introduction

Microelectrode arrays (MEAs) have been used in the field of bioelectronics as platforms to better comprehend how cells communicate in vivo [1,2,3,4] as well as in vitro [5,6,7,8]. For example, MEAs have been used to record the electrogenic activity of cardiomyocyte-like cells [9,10,11,12,13,14] and the release of neurotransmitters at the synaptic cleft of a neuron [15,16,17,18,19]. In addition, these platforms have been used to conduct toxin or drug screenings to monitor their effects on cultured cell-lines [7,20,21,22,23]. MEAs are traditionally made in a clean room where high-resolution features can be achieved. However, with the need for novel designs, cleanroom processing can become costly and time consuming, depending on the number of fabrication steps required to create the sensor. This is not ideal for rapidly prototyping different MEA layouts where multiple design iterations of a mask are required. Additive manufacturing on the other hand has shown to be advantageous when fabricating low-volume sensors in a time-efficient manner and with an overall lower material waste [24]. In particular, piezoelectric drop-on-demand (DOD) inkjet printing has attracted interest from the microfabrication community for its maskless design flexibility and its large variety of jettable materials encompassing metal nanoparticles [25], organometallics [26], polymers [26,27,28], dielectric materials [29], 2D materials such as graphene [30,31], semiconductor nanomaterials [24], nanowires [32,33,34], ceramics [35], carbon nanotubes [26], proteins [36,37], and even living cells [38]. With DOD inkjet printing, novel MEA designs have already been created for recording extracellular potentials of cells in vitro [15,39,40,41,42], as well as in vivo [43,44].

The call for recording in 3D has attracted a growing interest in the fields of organ-on-a-chip and bioelectronics [8,45,46,47,48]. As a response, 3D MEAs have been developed for in-vivo [49,50,51,52,53] and in-vitro [54,55,56] studies. Typically these 3D MEAs are produced in a clean room, as well as by subtractive processes such as photolithography in combination with dry and wet etching, wire-electrode cutting, bulk micromachining, and laser cutting [57,58,59]. However, this fabrication approach can become elaborate and expensive for small-volume production of novel 3D MEA designs, which may require varying heights to penetrate different layers of tissue. In addition, some established process steps have only been optimized for inert metals and silicon-based materials. This can also limit the possibilities of fabricating 3D electrodes with flexible and stretchable materials, which can better interface with soft tissue [57,58,59]. These issues could be addressed by using an additive manufacturing approach. However, most additive approaches use non-conductive materials to form the 3D structures, which later require conductive films to be deposited [59]. Alternatively, some designs have used 3D printing to define self-insulated microelectrode arrays [60] for future cell culture applications [61,62,63,64]. Even though these 3D MEAs can rapidly be produced in a cost-effective manner, they lack the lateral resolution needed to interface with individual cardiomyocyte or neuronal cells. 

Piezoelectric DOD inkjet printing has been used to print a number of materials and in particular conductive metal-based inks [25]. By controlling the ejection of individual droplets and the solvent evaporation of the ink, micron-sized 3D structures can be printed on demand with varying heights up to the mm range [65,66,67]. Alternatively, ultraviolet light can also be utilized between each layer of ink to form conductive 3D structures [68,69]. So far, a wide variety of shapes have been printed such as micropillar arrays, micro-helixes, micro-zigzags, and hexagonal and cactus structures [65,70]. However, the most common application for these inkjet printed 3D structures has been to use them as interconnects [70,71,72,73,74]. Currently, micropillars with a diameter as small as 22 µm have been fabricated using a commercial inkjet printer [72]. The lateral dimensions are in the same order of magnitude as cardiomyocyte cells, which could be interesting for future heart-on-a-chip and organ-on-a-chip systems.

This paper demonstrates the possibility of inkjet printing 3D MEAs for monitoring cardiomyoycte-like cells. A continuous printing strategy is introduced for higher-throughput production of 3D MEAs using silver nanoparticle ink. The growth and morphology of the printed and sintered 3D electrodes were investigated. In addition, a constant and pulsed electroplating method were compared on Ag 3D electrodes in order to reduce the leakage of cytotoxic silver ions into the electrolyte. Impedance spectroscopy and cyclic voltammetry were used to electrochemically characterize the 3D MEAs.

## 2. Materials and Methods

### 2.1. Printing 3D Microelectrode Arrays

3D microelectrode arrays (MEAs) were printed on a 125 μm thick polyethylene naphthalate (PEN) film (Teonex Q65HA, DuPont Teijin Films, Wilton, UK) using a silver nanoparticle ink (Silverjet DGP 40LT-15C, Sigma-Aldrich, St. Louis, MO, USA) with a state-of-the-art inkjet printer (CeraPrinter F-Series, Ceradrop, Limoges, France). Prior to printing, the Ag nanoparticle ink was allowed to equilibrate up to room temperature before sonication (Bransonic ultrasonic cleaner 5510E-MTH, Branson ultrasonics, Danbury, CT, USA) for 10 min, filtered using a poly(vinylidene fluoride) filter (GD/X, Whatman, Maidstone, UK; pore size: 0.2 μm), and loaded into a disposable 1 pL cartridge (DMC-11601, Fujifilm Dimatix, Santa Clara, CA, USA).

A unipolar voltage pulse of 40 V with a rise, dwell, and falling time of 3, 12, and 1 µs, respectively, was applied to the nozzle plate to eject individual ink droplets. The nozzle plate and the sample stage were held at 55 and 60 °C, respectively.

The feedlines of the 3D MEAs were printed at a frequency of 1 kHz with a drop spacing of 41.9 µm, with an individual Ag droplet diameter on the PEN film exhibiting ~60 µm. The 3D microelectrodes were printed using a moving print head with a measured drop-to-drop time interval of 4.1 s. Once printed, the Ag ink was dried on the sample stage and thermally sintered in an oven for 2 h at 150 °C. 

### 2.2. Electroplating Procedure

Gold and platinum were electrodeposited onto the 3D electrodes in order to inhibit direct contact of silver with the medium during cell culture experiments. In order to contain the electrolyte, glass rings (height, outer diameter, and inner diameter of 15, 17, and 14.6 mm, respectively) were bonded onto the printed arrays using 10:1 (*w*:*w*) polydimethylsiloxane (Sylgard 184 kit, Dow Corning, Wiesbaden, Germany), degassed for 20 min and cured for 30 min at 100 °C. 

An aqueous potassium gold cyanide bath (KAu[CN]_2_, Pur-A-Gold 401B, Enthone-OMI, Hertogenbosch, Netherlands) was used for Au electrodeposition. The KAu[CN]_2_ bath displayed a pH and conductivity of 5.8–5.85 and 10.1–10.2 S m^–1^, respectively, at room temperature (inoLab multi 9310 IDS, WTW, Weilheim in Oberbayern, Germany).

For the Pt electrodeposition, 60 mM hexachloroplatinic acid (H_2_PtCl_6_, MaTecK, Jülich, Germany) solution was prepared in ultrapure water containing 0.1 wt% sodium dodecyl sulfate (SDS ultrapure, neoLab Migge GmbH, Heidelberg, Germany). The pH and conductivity of the bath at room temperature were 1.0–1.3 and 3.02–3.04 S m^–1^, respectively.

Chronoamperometry was used to electroplate the 3D microelectrode arrays using a potentiostat (VSP-300, Bio-Logic Science Instruments, Seyssinet-Pariset, France) in a 3-electrode configuration. The printed structures acted as the working electrode, a larger platinum mesh as the counter electrode and a Ag/AgCl electrode (3 M NaCl, RE-6, BASi West Lafayette, IN, USA) was used as the reference. 

The reducing potential for KAu[CN]_2_ and H_2_PtCl_6_ was set to −1.15 V and −0.2 V vs. Ag/AgCl, respectively. Constant (CED) and pulsed (PED) voltage waveforms were applied to the 3D microelectrodes and later compared using the same effective deposition interval. 

### 2.3. Printing the Passivation

After the Au electrodeposition but before plating the sensors with Pt, an ultraviolet (UV) curable acrylate ink (DM-IN-7003-I, Dycotec Materials Ltd., Calne, UK) was used to passivate the feedlines whilst allowing the 3D microelectrodes to protrude through the insulation layer. A 10 pL cartridge (DMC-11610, Fujifilm Dimatix, Santa Clara, CA, USA) was filled with the UV curable acrylate ink which was passed through a 0.22 μm polyethersulfone (PES) filter (TPP, Trasadingen, Switzerland) and covered with Al foil to protect against light.

Prior to printing, the glass ring was carefully removed and the chips were plasma-activated (Femto, Diener electronic, Ebhausen, Germany) with oxygen at 30 W with a pressure of 0.3 mbar for 12 s, to enable better wetting of the ink on the substrate. The 3D MEAs were fixed to the inkjet printer’s substrate holder with Kapton tape (VWR, Darmstadt, Germany) covering the contact pads. A 40 µm spacing around the 3D electrodes was defined in the print layout to ensure the structures would not be covered in ink. The nozzles were manually aligned with the 3D microelectrodes using the printer’s on-board camera. A single layer of acrylate ink was printed with an ejection frequency of ~253 Hz and a drop spacing of 40 μm. A unipolar voltage pulse of 50 V, with rise, dwell, and falling times of 6.1, 10, and 1 μs, respectively, was applied to the nozzle plate to eject the ink droplets. During the printing process, the sample stage and the nozzle plate were both heated to 50 °C. The ink was lastly cured using an inbuilt UV lamp with an intensity of 5 W cm^−2^ at a sample stage velocity of 50 mm s^−1^ resulting in an approximate UV dose of 1 J cm^−2^. 

### 2.4. Focused Ion Beam and Scanning Electron Microscopy

A focused ion beam (FIB, Helios NanoLab 600i, FEI Deutschland GmbH, Frankfurt, Germany) sectioning comprising of a milling and polishing process was used to investigate the 3D microelectrode’s internal structure. The thermally sintered 3D microelectrode array (printed with 1606 droplets of silver nanoparticle ink) was firstly sputtered with Pt (45 s, 15 mA). A milling area of 40 × 11 μm^2^ around the pillar was targeted and a milling current of 9.3 and 2.5 nA was applied. Finally, the polishing step used a current of 2.5 nA before a scanning electron microscope (SEM, Magellan 400, FEI Deutschland GmbH, Frankfurt, Germany) was used to capture the internal structure. The diameter of the pores was calculated by counting the number of equivalent pixels and multiplying this by the scaling index 1 pixel = 0.541 nm.

Inkjet-printed samples were firstly sputtered with gold using a high vacuum coating system (5 × 10^−5^ bar, 40 s, 40 mA, approx. film thickness 10 nm; BAL-TEC Med 020, LabMakelaar Benelux BV, Zevenhuizen, The Netherlands) before imaging them in the scanning electron microscope (JSM-6060LV, JEOL, Tokyo, Japan). A conductive double-sided carbon pad and copper tape was used to fix the substrate on the holder and prevent charge accumulation. The 3D electrodes were imaged at varying acceleration voltages, magnifications, and substrate tilts. All of the images captured were later tilt-corrected using GIMP.

### 2.5. Electrochemical Characterisation and Data Analysis of 3D Electrodes

Cyclic voltammetry and impedance spectroscopy were used to characterize the 3D electrodes, using a potentiostat (VSP-300, Bio-Logic Science Instruments, Seyssinet-Pariset, France). Both techniques used phosphate-buffered saline (Modified Dulbecco’s PBS, Sigma-Aldrich) as the electrolyte. Cyclic voltammetry was performed in a 3-electrode configuration, over a potential range of −0.4 to 0.8 V vs. Ag/AgCl (3 M NaCl), and with a scan rate of 50 mV s^−1^. Impedance spectroscopy measurements were carried out in a 2-electrode setup. A Pt wire was used as a combined reference and counter electrode and individual 3D microelectrodes were set as the working electrode. No bias voltage was applied against the open circuit potential. A sinusoidal waveform with an amplitude of 10 mV vs. the open circuit potential was used to measure the impedance of the electrodes with a frequency range of 10^0^–10^4^ Hz.

The current integration in the cyclic voltammograms used background subtraction with a linear fit to remove the influence of capacitive currents and electrolyte resistance. The data points before the oxidation and reduction peaks were used for the fit in the voltage range of −0.3 to 0 V and 0.7 to 0.4 V, respectively. Thereafter, the charge was back-calculated by summing all data points above 0 A (oxidation peaks) and below 0 A (reduction peaks), and multiplying by the respective sampling time interval. For comparison, both of the charges during the cathodic and anodic reactions are displayed separately as positive values.

### 2.6. Laser Profilometry and Data Analysis

A 3D laser scanning confocal microscope (VK-X250, Keyence, Osaka, Japan) in combination with a 50× objective (50×/0.95 CF Plan Apo OFN25, Nikon, Japan) was used to measure the growth of the inkjet-printed pillars. Each pillar was individually scanned using two laser intensities (double-scan feature) to better capture the morphology of the pillars. The microscope’s neutral-density filter and the laser’s brightness were set automatically using the device’s auto gain function. All measurements were taken with a *z*-pitch of 100 nm, on a vibration-dampened table (Vision IsoStation, Newport, RI, USA) to reduce external interferences.

Profilometric data was processed with a custom MATLAB script. The background area for each pillar was manually defined and fitted using a two-dimensional polynomial of order 1 in both *x* and *y*. After subtracting the background, a median filter of kernel size 3 × 3 was applied to remove any remaining high-frequency artifacts. The absolute maximum of the *z*-data was defined as the pillars’ height. Horizontal and vertical profiles were taken to evaluate the width and tip width of the pillars. The width of each individual pillar was defined as the distance closest to 50% of its maximum height. The pillar tip width was defined as the diameter closest to 500 nm below its maximum height. The pillar width measured in the *x*- and *y*-profile was averaged. This average was the final value for the pillar width. The same process was performed for the pillar tip width.

### 2.7. HL-1 Cell Culture and Measurement of Extracellular Signals

Cell culture materials and chemicals such as _L_-glutamine, fetal bovine serum, and penicillin/streptomycin were purchased from ThermoFisher Scientific (Waltham, MA, USA). Additionally, Claycomb medium, trypsin-EDTA solution, norepinephrine bitartrate, fibronectin, and gelatin were bought from Sigma-Aldrich (St. Louis, MO, USA). Finally, ascorbic acid, ethanol (≥99.5%), and 2-propanol (≥99.5%) were purchased from Carl Roth (Karlsruhe, Germany). An Ultra Clear purification system (EvoquaWater Technologies, Barsbüttel, Germany) was used to produce the required deionized water.

HL-1 cells were cultured in Claycomb medium supplemented with penicillin/streptomycin (100 U mL^−1^ and 100 μg mL^−1^), _L_-glutamine (2 × 10^−3^ M), fetal bovine serum (10%), and norepinephrine (0.1 × 10^−3^ M). Thereafter, the cells were placed in a humidified incubator (CB210 CO2, Binder, Tuttlingen, Germany) set at 37 °C in an atmosphere of 5% CO_2_. Once the cell line reached confluency and observable mechanical contractions, the cells were detached via incubation in a protease solution (0.05% Trypsin-EDTA) and used for the experiments. The 3D microelectrode array (3D MEA) chips were oxygen-plasma treated (0.8 mbar, 80 W, 5 min, Diener Femto, Diener electronic, Ebhausen, Germany) and sterilized by dipping into 2-propanol prior to cell seeding. Once the chips were dry, they were incubated with a solution of fibronectin (5 μg mL^−1^) and gelatin (0.2 mg mL^−1^) for ~1 h at 37 °C. The protein solution was removed and the 3D MEA chips were rinsed with Dulbecco’s phosphate-buffered saline (D8662, Sigma-Aldrich). The HL-1 cells were then seeded onto the chips and ~3 days *in vitro* were required to reach confluency. An inverted microscope (Axiovert 40 CFL, Carl Zeiss, Oberkochen, Germany) with a 5× objective was used to optically evaluate the confluent HL-1 cell layer.

Once a confluent HL-1 cell layer was observed on the 3D electrodes, extracellular signals were recorded amperometrically using a custom-built 64-channel amplifier in a grounded Faraday cage. Detailed information regarding the amplifier can be found in previous studies [17,75]. The amplifier system was set to have an active bandwidth of 1 mHz to 3.4 kHz with a sampling rate of 10 kHz per channel, and an output signal amplification of 1 pA mV^−1^. A single Ag/AgCl (3 M NaCl, RE-6, BASi, West Lafayette, OH, USA) reference electrode was connected to the medium to close the electrochemical circuit. The cell culture medium was exchanged with an initial volume of 1 mL on the chip 1 h prior to the start of the experiment. During the experiment, freshly prepared 5 mM norepinephrine solution (dissolved in 33.6 mM ascorbic acid) was added in 5 μL portions to chemically stimulate the cells until induced cellular activity was perceived. To confirm the cellular origin of the extracellular potential signals, 100 μL of 1 M SDS was injected into the medium. On average, the total measurement time of the experiment was 5 min.

### 2.8. Data Analysis of Extracellular Potential Signals from HL-1 Cells

Each active channel was individually examined using MATLAB to analyze the extracellular potential recordings of the HL-1 cells. A first-order polynomial fit was subtracted from each individual channel to remove baseline fluctuations. Spikes with a minimum peak prominence of 150 pA were flagged. Individual spikes were cropped from the original trace starting 100 ms before and after the peak. Finally, the average and standard deviation of all field potential spikes of a given trace were calculated for display.

## 3. Results

### 3.1. Formation of 3D Microelectrodes

#### 3.1.1. Pillar Growth Using a Continuous Printing Method

**Figure 1:**
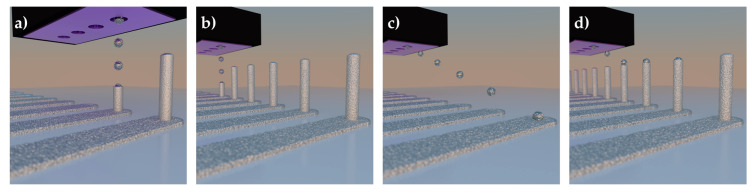
3D illustrations of the two methods for printing 3D microelectrode arrays: (**a**,**b**) stationary and (**c**,**d**) continuous.

The growth of pillar structures using pico-liter droplets of Ag nanoparticle (AgNP) ink was investigated for printing 3D microelectrode arrays (MEAs). Two different printing methods were initially investigated, as illustrated in Figure 1. The first method deposited individual droplets of ink in a stationary environment, to control alignment and ejection frequency for droplet drying, which are key for stacking the AgNP ink into a 3D pillar [70]. Due to the droplets’ small volume, surface tension has a much larger influence than gravitational forces (low Bond number), allowing the formation of 3D structures without the need for intermediate layer sintering stages [75]. However, a significant issue of a stationary system is the throughput of this method, as this allows only one pillar to be printed at a time. This is not ideal for a high throughput production of 3D microelectrode arrays. Therefore, the second method utilized a moving print head to allow multiple pillars to be fabricated in parallel. 

**Figure 2:**
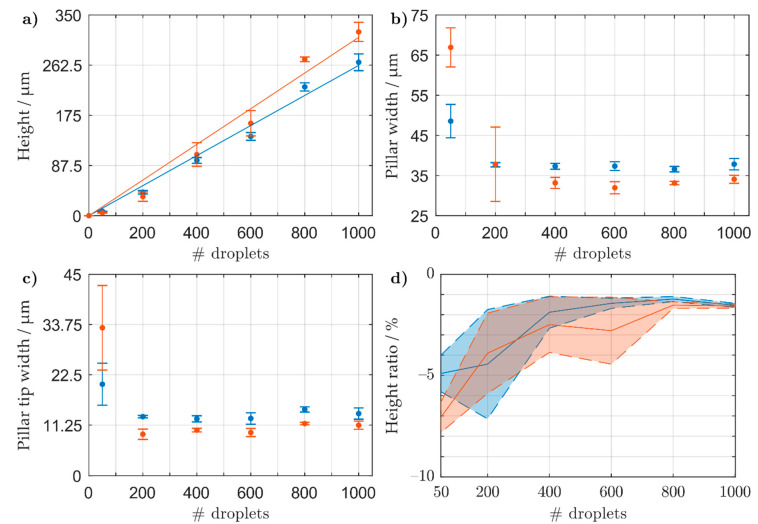
Profilometric data of unsintered pillars printed with a drop to drop (DtoD) delay of 4.1 (red) and 7.5 s (blue) on a heated sample stage (60 °C) and nozzle plate (55 °C). 50 to 1000 droplets of AgNP ink were printed and the pillar’s (**a**) height, (**b**) pillar width, and (**c**) pillar tip width were measured. (**d**) Relative change in height between sintered and unsintered pillars for both DtoD delays. The average height reduction ratio (solid lines) and standard deviation (dashed lines and shaded areas) for each number of droplets was calculated using 8 pillars. A linear fit with a set *y*-intercept at a height of zero was applied to (a) showing an incline of 262 ± 6 nm droplet^−1^ (blue) and 311 ± 11 nm droplet^−1^ (red).

From the initial results shown in Figure S1 (Supplementary Material), a continuous printing method exhibited a consistent stacking of material with a drop-to-drop (DtoD) delay between 4–8 s. In particular, DtoD delays of 4.1 and 7.5 s were further investigated, as they both exhibited exemplary values in the optimal range as shown in Figure S1d. In Figure 2a, both DtoD delays show a constant growth with increasing droplet numbers. When applying a linear fit to the data, a slope of 311 ± 11 nm droplet^−1^ (confidence level: 95%) and 262 ± 6 nm droplet^−1^ (confidence level: 95%) for DtoD time intervals of 4.1 and 7.5 s were calculated, respectively. Both DtoD delays demonstrate a similar initial growth, however, at higher droplet numbers the shorter DtoD delay exhibits taller pillars. For a DtoD delay of 4.1 and 7.5 s, an average pillar tip width of 11 ± 1 and 14 ± 1 μm, and an average pillar width of 33 ± 1 and 37 ± 1 μm were measured, respectively. For a DtoD delay of 4.1 s, a higher velocity of the print head was used, which we assume encourages faster evaporation of the droplet’s solvent. This confines the nanoparticles in a smaller area to form narrower and taller pillars. To confirm the linear fit shown in Figure 2a, 3D MEAs were printed with 321, 643, and 964 droplets, which correspond to heights of 100, 200, and 300 μm, respectively for a DtoD delay of 4.1 s. The structures were imaged under a scanning electron microscope (SEM), shown in Figure S2, displaying the expected pillar heights.

#### 3.1.2. Thermal Sintering of 3D Microelectrodes

**Figure 3:**
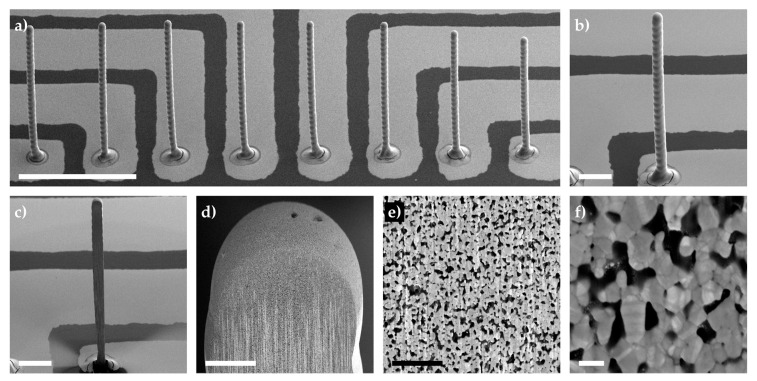
Tilt-corrected scanning electron microscope images of (**a**) a sintered 3D microelectrode array, (**b**) a pillar under focused ion beam analysis, and (**c**) a pillar after bulk milling and polishing. Different magnifications of the pillar shown in (**c**) are shown in (**d**–**f**). All pillars were printed with approx. 1600 droplets of AgNP ink, displaying a width of ~32 μm and a height of 500–560 μm. All images were captured using an acceleration voltage of 3 kV and a substrate tilted at 52–54°. Scale bars represent (**a**) 500 μm, (**b**) and (**c**) 100 μm, (**d**) 10 μm, (**e**) 1 μm, and (**f**) 100 nm.

After printing the 3D electrodes with varying droplet numbers, they were placed in an oven at 150 °C for 2 h for sintering. The relative change in height for each individual pillar was measured and the average and standard deviation are shown in Figure 2d for a drop-to-drop (DtoD) delay of 4.1 (red) and 7.5 s (blue). The relative change in height was small, with a larger decrease in pillar height at low droplet numbers. At 50 droplets, both delays showed a relative change in height of −5% to −7%, whilst at higher droplet numbers the relative change was lower: ~−1.5%. To better understand what occurred inside the printed and thermally sintered electrodes, a focused ion beam (FIB) cut was applied to a tall 3D microelectrode (array and pillar under investigation are shown in Figure 3a,b, respectively). After applying an initial cut and a polishing step (Figure 3c), the internal structure of the pillar was imaged with varying magnifications (Figure 3d–f). In the captured images, it can be seen that the 3D electrode’s internal structure is porous with pore sizes around 79 ± 32 nm (*n* = 24, measured from Figure 3f). This, in turn, explains why the relative change in the pillars’ height after sintering does not drastically decrease (shown in Figure 3d) as the nanoparticles do not form a bulk material.

### 3.2. Electrochemically Stable 3D Microelectrodes: An Electroplating Approach

#### 3.2.1. Constant vs. Pulsed Electrodeposition

**Figure 4:**
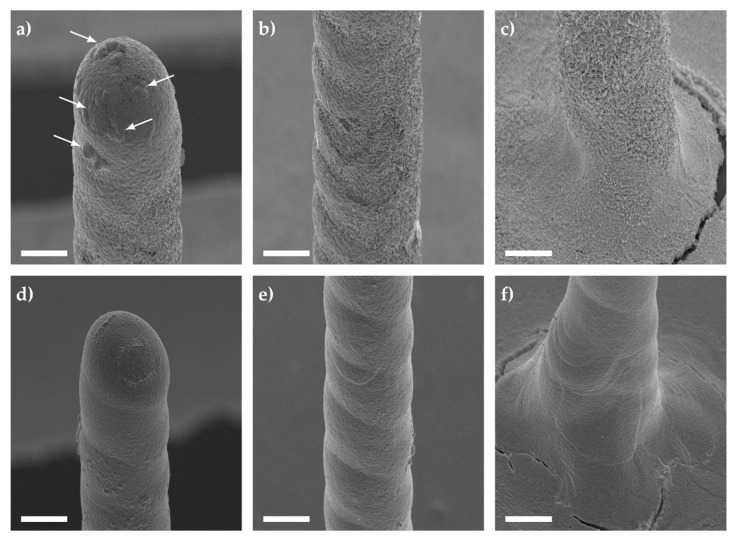
Tilt-corrected scanning electron microscope images of Au galvanized pillars using (**a**–**c**) constant and (**d**–**f**) pulsed electrodeposition. A constant potential of −1.15 V was applied for 300 s in comparison to a pulsed potential (−1.15 V for 20 ms, 0.4 V for 70 ms, 0 V for 10 ms vs. Ag/AgCl (3 M NaCl) reference electrode) with 15,000 cycles. The tip (**a**,**d**), middle (**b**,**e**), and base (**c**,**f**) were imaged using an acceleration voltage of 15 kV, a magnification of 1000×, and a substrate tilted at 45°. The scale bars shown in (**a**–**f**) correspond to a length of 20 μm.

To better understand which electroplating method would be more suitable for printed structures, constant (CED) and pulsed (PED) electrodeposition protocols were compared. The CED protocol was tested using an electrodeposition potential of −1.15 V vs. Ag/AgCl (3 M NaCl) for a deposition interval of 300 s. This was compared to the PED protocol, which applied −1.15 V for 20 ms followed by 0.4 V for 70 ms before setting the electrode to 0 V for 10 ms vs. Ag/AgCl (3 M NaCl). This was repeated 15,000 times. The resulting galvanized structures can be seen in Figure 4. 

For the CED protocol, semi-spherical bulges (shown with arrows) and interlaced Au spikes were observed at various sections of the 3D electrode (see Figure 4a–c). In contrast, the PED protocol displayed a smoother deposition of Au (see Figure 4d–f), in particular when we compare Figure 4b,e. The varying roughness of gold deposits can be affected by many factors, such as additives in the electrodeposition bath, the working electrode’s initial crystalline morphology, diffusion, the electric field, and finally the applied potential. For instance, if the reduction potential is near the required potential to split water molecules, this could lead to the formation of hydrogen bubbles forming nanostructured platelets [76]. In order to negate the roughness and possible hydrogen embrittlement [77,78,79], a PED protocol can be used which modifies the Nernst diffusion layer [79]. At the immediate vicinity of the working electrode, the concentration of Au ions fluctuates with the frequency of the applied pulses [79]. This allows the formation of finer grain deposits by intermittently delivering a short pulse with a high current density, resulting in a higher nucleation rate [79]. In order to quantify the inertness of the gold coated electrodes (either by CED or PED), they were stimulated in electrolyte solution similar to the cellular medium.

#### 3.2.2. Electrochemical Characterization of Au Electroplated 3D Electrodes

**Figure 5:**
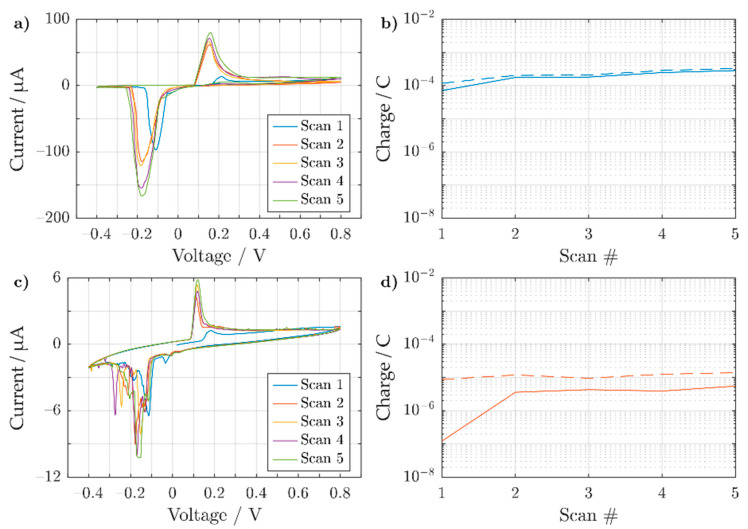
Cyclic voltammogram showing the 5 sweeping cycles for (**a**) sintered Ag and (**c**) Au-coated 3D electrode using the CED protocol for 300 s, both in phosphate-buffered saline. Both 3D electrodes have an exposed height of ~450 μm in electrolyte. Both pillars were swept from −0.4 to 0.8 V vs. Ag/AgCl (3M NaCl), with a scan rate of 50 mV s^−1^. The accumulated charge for both the oxidation (solid line) and reduction (dashed line) peaks are shown for (**b**) sintered Ag and (**d**) Au coated 3D electrode.

After electroplating the 3D electrode array, a cyclic sweep in phosphate-buffered saline (PBS) was conducted to evaluate the inertness of the deposited Au layer. To properly evaluate the electrodes, the feedlines were passivated, allowing only the 3D structures to protrude (*h*~450 μm). The cyclic response for a bare silver and a galvanized 3D electrode was compared, as shown in Figure 5. 

Oxidation and reduction peaks were found on the silver 3D electrode (shown in Figure 5a around 149 ± 4 mV and −177 ± 3 mV vs. Ag/AgCl (3M NaCl), respectively (values averaged over scan 3–5). The most probable reason for the peaks’ existence is due the buffer’s high chloride concentration, which allows the redox reaction to occur.

Ag + Cl^–^ ⇋ AgCl + e^–^ (1)

Both the oxidation and reduction peaks shown in Figure 5a are far apart from one another, which indicates a kinetically limited system. The slow kinetics could be attributed to the formation of AgCl on silver, which follows a complex adsorption-desorption mechanism [80]. Similar peaks have also been found in literature when cycling a silver wire in phosphate-buffered saline [81]. With increasing scan number, there is an increase in the peak amplitude correlating to an increase in oxidized and reduced species. By integrating over individual redox peaks, the charge for each peak can be calculated for every cycle, as shown in Figure 5b. Here, the reduction of AgCl to form Ag is slightly more prominent than its counter reaction. This would suggest that the silver 3D microelectrode does not degrade. However, over prolonged cycling, more Ag becomes oxidized and then reduced, as shown by the general increase in charge over the scan number. If the formed AgCl cannot be reduced back into Ag, the 3D microelectrode could eventually degrade.

As a comparison to the 3D Ag electrode, a similar Au-coated electrode (using the CED protocol for 300 s) was cycled in PBS, as shown in Figure 5c. In comparison to Figure 5a, the oxidation peak at 115 ± 1 mV vs. Ag/AgCl (3M NaCl) (averaged over scan 3–5) was notably smaller. For the reduction, however, several peaks were observed between 0 and −0.35 V with the lowest appearing at −153 ± 9 mV vs. Ag/AgCl (3M NaCl) (averaged over scans 3–5). The wider backward scan peaks can be attributed to a hindered reduction of AgCl with the onset of O_2_ reduction due to the lack of nitrogen purging. A similarly hindered reduction of AgCl peaks in PBS has also been found in the literature [82]. As the exposed surface of the electrode was modified with an inert metal, ideally no silver oxidation or reduction peaks should be present. However, the first scan already shows a small Ag oxidation peak that increases with increasing scan number. It is possible that the underlining Ag structure was still electrochemically available, which could be explained by interdiffusion of the two materials [83,84]. Comparing Figure 5b,d, however, the amount of charge transferred was two orders of magnitude lower in the case of the gold plated structure. In addition, the charge remains stable over consecutive cycles. However, the reduction charge is higher than for the oxidation process. Most likely, this can be related to the onset of oxygen reduction, as the electrolyte was not purged with nitrogen prior to the experiment. In the case of the bare Ag pillars (Figure 5a), this process was masked by the overall larger current amplitudes.

**Figure 6:**
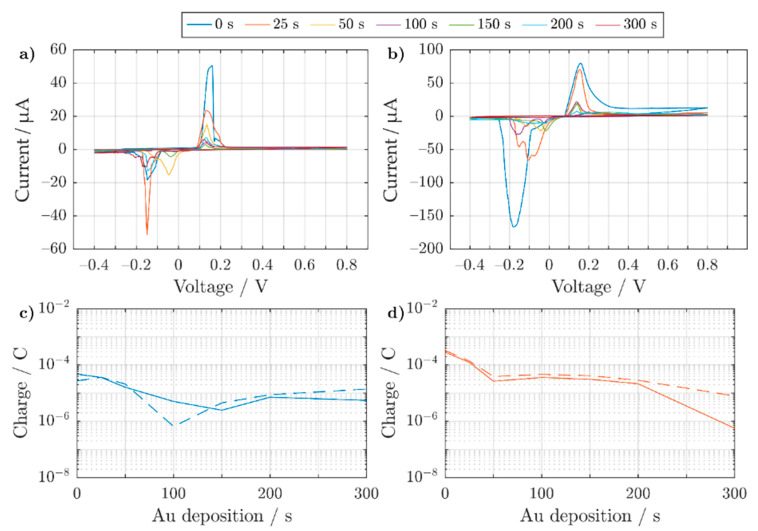
Cyclic voltammograms showing the 5th cycle response of Au galvanized 3D electrodes in PBS, using (**a**) constant and (**b**) pulsed electrodeposition. All 3D electrodes were of similar size with an exposed height of ~450 μm in electrolyte. Each trace represents an electrode, which was Au coated for a different effective deposition interval from 0 to 300 s. The same scan rate of 50 mV s^−1^ was used for each trace. (**c**,**d**) show the back-calculated charge for oxidative (solid lines) or reductive (dashed lines) processes occurring after constant and pulsed electrodeposition, respectively.

Coating the silver electrodes with gold lowers the availability of Ag ions to the electrolyte. In order to evaluate the better electroplating procedure to reduce the presence of exposed Ag, the response of electroplated electrodes (using a CED and a PED protocol) in phosphate-buffered saline are compared (see Figure 6). For increasing electrodeposition intervals, both protocols show a decrease in the oxidation and reduction peaks (see Figure 6a,b), which relates to a lower presence of exposed Ag. In order to better quantify the available presence of silver ions after electroplating, we calculated the transferred charge during the oxidative and reductive peaks as shown in Figure 6c,d. After a PED protocol, the charge associated with the oxidation process decreased when pillars were coated for prolonged time. As the internal structure of the electrodes is porous (see Figure 3), with a PED protocol the internal pores are steadily filled as opposed to a CED protocol (view Figure S3) [85]. However, the PED protocol also requires a longer deposition interval in order to properly coat the electrodes with finer Au grain deposits [79]. 

#### 3.2.3. Electrochemical Characterization of Pt Electroplated 3D Electrodes

As discussed above, plain electroplating of Au onto a silver electrode does not completely prevent the occurrence of Ag redox peaks in phosphate-buffered saline. To mitigate this, an additional fabrication step is required. Since Pt is a good conductor and is electrochemically inert, it is an ideal material to record or stimulate interfacing biological tissues or cells. However, the most commonly-used electroplating Pt baths use hexachloroplatanic acid (H_2_PtCl_6_) [86] which is strongly acidic. This causes metal delamination when directly electroplating onto silver electrodes (see Figures S5 and S6). Therefore, Pt was electroplated onto an existing Au coated 3D electrode.

**Figure 7:**
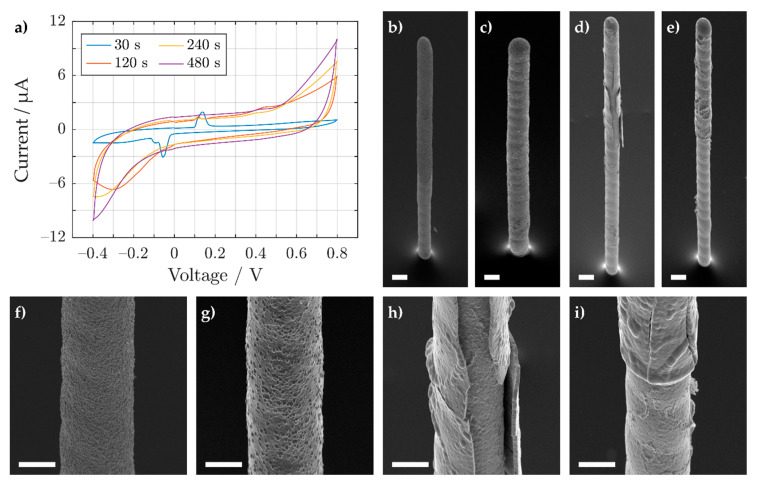
(**a**) Cyclic voltammogram showing the 5th cycle response of Ag–Au–Pt 3D electrodes in PBS. Each trace represents an electrode, which was first coated with Au for an effective deposition interval of 200 s and then further coated using Pt for an effective reduction time of 30, 120, 240, and 480 s (both used a PED protocol for electroplating). After cyclic voltammetry, tilt-corrected SEM pictures of the same 3D electrodes with different Pt deposition times are shown: 30 s (**b**,**f**), 120 s (**c**,**g**), 240 s (**d**,**h**), and 480 s (**e**,**i**). A magnification of 180× and 1000× was used for (**b**–**e**) and (**f**–**i**), respectively. All images used an acceleration voltage of 15 kV and a substrate tilted to 45°. The scale bar shown in (**b**–**e**) and the scale bar shown in (**f**–**i**) correspond to a length of 40 and 20 μm, respectively.

A pulsed electrodeposition results in a finer grain formation along the 3D electrode. Therefore, Pt was electrodeposited using a PED protocol, where each pulse consisted of a reduction held at −200 mV vs. Ag/AgCl (3 M NaCl) for 20 ms and an equilibrium phase for 80 ms at 0 V vs. the electrode’s open-circuit potential. In total 1500, 6000, 12,000, and 24,000 pulses were applied to the 3D electrodes, which resulted in an effective deposition interval of 30, 120, 240, and 480 s, respectively. The final Ag–Au–Pt electrodes were electrochemically characterized using cyclic voltammetry and imaged under the SEM, as shown in Figure 7. 

For an effective Pt deposition time of 30 s (blue trace), clear redox peaks for Ag remain visible in the cyclic voltammogram (Figure 7a). However, at longer deposition intervals (view purple trace) the peaks cannot be seen anymore, suggesting a reduced presence of silver. Under the SEM, the different 3D electrodes exhibit visible changes in the 3D electrodes’ morphology (compare Figure 7b–e). When Pt was electrodeposited for 30 s onto the electrode, a change in the 3D electrode’s roughness appeared at the tip and middle of the pillar (see Figure 7b). An enlarged view of the roughness at the midsection of the pillar is shown in Figure 7f. At the lower part of the electrode, however, there seems to be no apparent change in the pillar’s roughness. This suggests that the lower end of the pillar was not coated with Pt, allowing the Ag ions to diffuse past the Au layer. At 120 s the entire 3D electrode was fully covered with Pt as shown in Figure 7d. A magnified image of the midsection of the pillar is shown in Figure 7g. From 240 s onwards, however, cracking and delamination of the coated metal becomes apparent as shown in Figure 7h,i. This can likely be attributed to hydrogen entrapment and consequent stress for thicker Pt layers [87]. Even after delamination occurred, platinum continued to be deposited on the exposed electrode for longer electrodeposition times [87]. This can avoid the occurrence of Ag redox peaks in the presence of phosphate-buffered saline.

### 3.3. 3D Electrodes for Cell Culture

**Figure 8:**
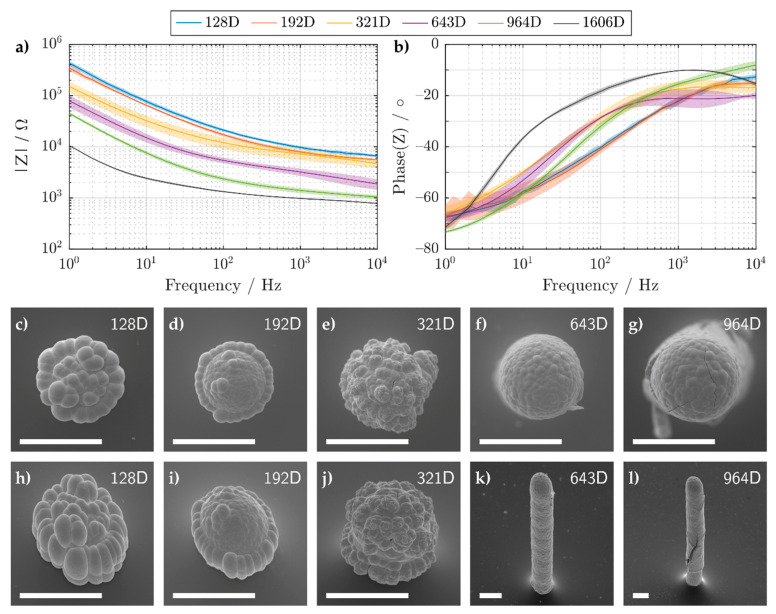
(**a**) Magnitude and (**b**) phase of the impedance of electroplated Ag-Au-Pt 3D microelectrodes, which were printed with different droplet numbers. The mean and standard deviation (solid line and shaded area, respectively) were calculated using 4 samples. (**c**–**l**) Tilt-corrected SEM images of the same 3D microelectrodes taken at a substrate tilt of (**c**–**g**) 0° and (**h**–**l**) 45°. All SEM images used an acceleration voltage of 15 kV. A magnification of (**c**–**j**) 1000×, (**k**) 350×, and (**l**) 250× was used. All scale bars shown have a length of 40 μm. The numbers in the legend and the upper right corner of the SEM images correspond to the amount of ejected Ag nanoparticle droplets.

Electrochemical impedance spectroscopy was used to characterize the 3D electrodes’ suitability for cell recordings. To this end, electrodes with varying heights were printed and electroplated with Au and Pt (fabrication method shown in the Supporting Information). Their impedances measured in PBS, along with corresponding SEM images, are shown in Figure 8. Depending on the droplet number, either semi-spherical or pillar structures can be fabricated. For droplet numbers below 643D, a smaller electrode opening was formed after passivation. After the Pt galvanization process, cauliflower patterns are visible, which arise due to overlapping primary diffusion zones [88]. 

As we wished to record cellular activity, we required a low enough impedance whilst also maintaining spatial selectivity. In Figure 8a, the absolute impedance of the printed and galvanized 3D electrodes decreases with increasing surface area. This results in the spreading resistance (also known as the electrolyte resistance) becoming the dominating impedance at higher frequencies. The spreading resistance defines the lowest resistance you can have for a certain electrode geometry. For example, this resistance can theoretically be calculated for semi-spherical microelectrodes with the following equation:

(2) $$R=\frac{\rho }{2\pi r}\;\;,$$

where *ρ* is the resistivity of the electrolyte and *r* the radius of the half-sphere microelectrode. Using Equation (2), a half-sphere microelectrode with a radius of 20 μm and a measured PBS conductivity of 1.58 S m^−1^, yields a spreading resistance *R* of ~5 kΩ. This value is comparable to the measured impedance of 3D electrodes printed with 192 and 321 droplets, which at 10 kHz exhibited a resistance of 5.5 ± 0.5 and 5 ± 1 kΩ, respectively. However, for 3D electrodes printed with 128 droplets, a resistance of 6.5 ± 0.5 kΩ was measured. The higher resistance can be explained by the flatter morphology of the electrode (see Figure 8c,h) which does not fully resemble a half-sphere nor a planar disk ($R_{disk}=\frac{\rho }{4r}=\;\sim 8\;kΩ)$.

For frequencies below 10 Hz, the impedance was not significantly affected by the spreading resistance. In this regime, all impedance curves could be fitted by a single constant phase element (see Table 1) indicating deviations from an ideal capacitor (α~0.7). The low impedance values were later exploited in order to record cellular activity of HL-1 cells. 

### 3.4. Extracellular Potential Recording of HL-1 Cells Using Inkjet-Printed 3D Electrodes

**Figure 9:**
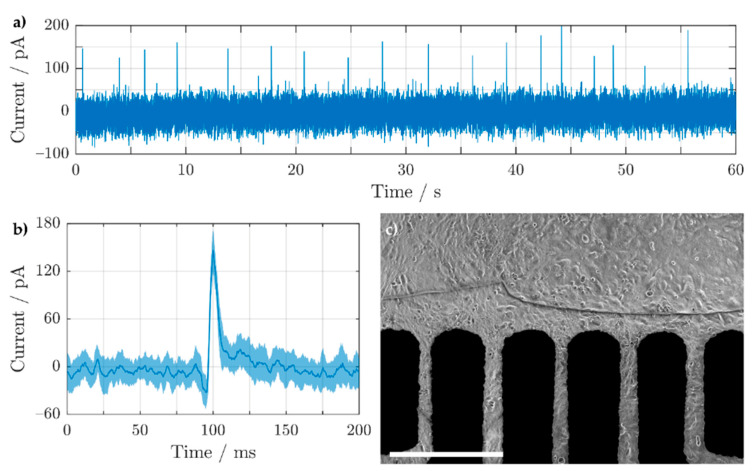
(**a**) Extracellular potential recording of HL-1 cells on a Ag-Au-Pt 3D MEA recorded using an in-house-built amplifier. (**b**) The mean and standard deviation (solid line and shaded area, respectively) of the extracellular potentials are displayed in (**a**). (**c**) Optical image of the confluent HL-1 cells taken from the backside of the MEA using an inverted microscope (5× objective), with a 500 μm scale bar.

In order to test the 3D electrodes’ biocompatibility and functionality for future cell experiments, cardiomyocyte-like cells (HL-1) were cultivated on similar 3D electrodes, as shown in Figure 8e,j. This electrode size was chosen as it exhibited a low enough amplifier noise, while still maintaining a prominent 3D shape. This was due to the recording setup, which measured the extracellular potentials of the HL-1 cells amperometrically, by exploiting the capacitive nature of the electrodes (view Figure S7 for more information). After 3 days *in vitro*, the cells reached confluency and extracellular recordings were performed as shown in Figure 9. The extracellular activity of the HL-1 cells was induced using norepinephrine and later stopped by injecting sodium dodecyl sulfate (SDS), which kills the cells. In order to avoid interfering Faradaic reactions (e.g., oxidation of norepinephrine [89]), a bias potential of 0 V vs. Ag/AgCl (3 M NaCl) was applied. The spiking activity of the cells is shown in Figure 9a and the average and standard deviation of the extracellular potentials are displayed in Figure 9b. Finally, Figure 9c shows the confluent cell layer surrounding the electrodes where the extracellular potentials were measured.

## 4. Discussion 

Overall, the approach presented here allows the rapid prototyping of 3D MEAs, which can later be functionalized for cell culture applications. Silver nanoparticle ink was used to produce the 3D electrodes due to its established printing stability, commercial availability, the material’s high conductivity (after post-treatment), and the low cost required for making prototypes. The successive electrodeposition of Au and Pt inhibited the exposure of Ag ions to the electrolyte solution. In addition, HL-1 cells were cultured on top of the Ag-Au-Pt 3D MEAs for extracellular experiments. Nevertheless, the Pt electrodeposition should be improved to prevent delamination of the 3D structures. Lowering the reduction potential to remove internal hydrogen stress, increasing the temperature for improved mass transport of Pt ions, or reducing the pulse duration to form finer deposited grains would be viable strategies in this context.

As the underlining silver structure is not ideal for interfacing with cells, an alternative strategy would be to print directly with inert metal nanoparticle inks made of Au or Pt. These inks however are more expensive and often less reliable concerning the printing process in comparison to silver nanoparticle-based inks. For this reason, these inks are rarely used in low-cost applications. In addition, there is a need for future designs to incorporate flexible or stretchable materials which mimic the same Young’s modulus as cells [90]. Au and Pt nanoparticles typically require higher sintering temperatures in comparison to Ag nanoparticles. This increase in temperature is not ideal as it may induce irreversible stress in the soft films. Therefore, future investigations should also focus on implementing alternative curing methods such as photonic, selective laser, or infrared sintering, to allow inert and conductive structures on temperature sensitive substrates [91]. 

**Figure 10:**
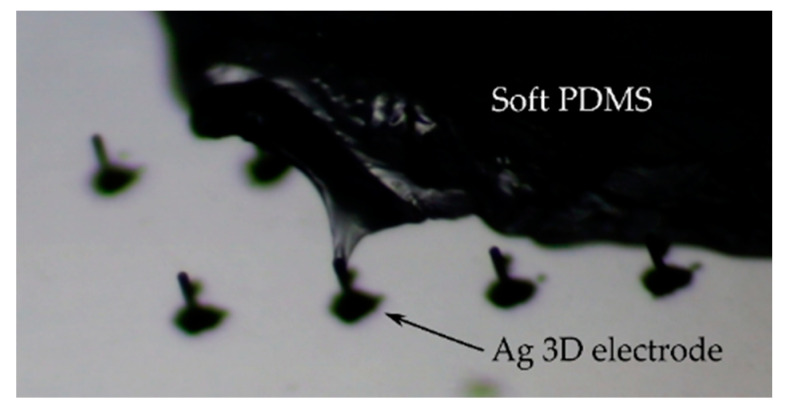
Silver 3D electrodes (sintered at ~220 °C) with a width and height ~30 µm and ~300 µm penetrating into soft PDMS (Sylgard 527, Dow Corning).

There are a number of applications in vitro and in vivo where 3D structures are required to penetrate soft tissues [57,59]. Therefore, alongside alternative curing methods, the mechanical stability of the printed 3D electrodes should also be examined in future studies. As proof-of-concept, Figure 10 shows a sintered Ag 3D electrode penetrating into a soft silicone. Overall, the use of inkjet printing is a powerful additive manufacturing technique, which we believe can have a positive influence on the fabrication of novel 3D sensors in bioelectronics.

## 5. Conclusions

We have demonstrated the fabrication of inkjet-printed and galvanized 3D microelectrodes for measuring local electrical activity of cardiomyocyte-like cells. A continuous printing method was investigated with respect to the drop-to-drop time interval to increase the throughput for the production of 3D MEAs. Standard 3D electrode designs had a diameter of ~30 µm and a height of up to ~560 µm. The external and internal structure of the printed electrodes was analyzed using confocal laser profilometry and scanning electron microscopy. Printed silver electrodes were galvanized with gold and platinum to prevent leakage of silver ions into the cell culture medium. Pulsed electrodeposition (PED) resulted in a much finer grain formation, compared to constant potential electrodeposition (CED). By applying an increasing number of pulses in succession, the presence of Ag ions diffusing towards the electrolyte was considerably decreased. Impedance spectroscopy and cyclic voltammetry were performed in phosphate-buffered saline to evaluate the double layer and electrochemical reactions of the 3D electrodes in solution. We demonstrated the possibility of recording extracellular activity from cardiomyocyte-like cells as a preliminary application in bioelectronics. Overall, we believe the approach presented offers a cost-effective alternative for rapidly prototyping 3D MEAs with potential future applications for stimulating or recording in organoid cultures. 

## Figures and Tables

**Table 1:** Constant phase element values for printed and galvanized electrodes below 10 Hz.

Droplet Num.	128	192	321	643	964	1606
*Q*/µFs^(α−1)^	0.7 ± 0.1	0.9 ± 0.2	2.3 ± 0.7	4 ± 1	5.7 ± 0.2	33 ± 4
α	0.71 ± 0.03	0.69 ± 0.05	0.65 ± 0.01	0.68 ± 0.02	0.77 ± 0.01	0.64 ± 0.05

## Data Availability

Not applicable.

## Supplementary Materials

The following are available online at https://www.mdpi.com/article/10.3390/s21123981/s1, Figure S1: Profilometric data of unsintered pillars printed at different drop-to-drop time intervals with a nozzle plate and sample stage held at 55 and 60 °C, respectively. Stationary inkjet printing (blue) and continuous inkjet printing (red) methods are shown for 3D pillars printed with (a–c) 50 and (d–f) 200 droplets of AgNP ink. (a,d) show the height, (b,e) the pillar tip width, and (c,f) the pillar width. 8–9 pillars were used to calculate the average and the standard deviation shown for blue and red, Figure S2: Tilt-corrected scanning electron microscope images of printed and sintered 3D microelectrodes with (a) 321 droplets, (b) 643 droplets, and (c) 964 droplets of AgNP ink. All 3 images were taken using an accelerating voltage of 15 kV, with a magnification of 180×, and a substrate tilted at 45°. The scale bars shown in all images correspond to a length of 100 μm, Figure S3: Optical images showing (a,c) top, and (b,d) underside of Au electroplated silver 3D MEAs. (a,b) were electroplated using a constant potential and (c,d) using a pulsed potential. The effective deposition time of Au was increased from left to right 0, 25, 50, 100, 150, 250, 250, and 300 s (1–8, respectively). The scale bar shown in each picture corresponds to a length of 1 mm, Figure S4: Tilt-corrected scanning electron microscope images of thermally sintered pillars printed with (a,c,d) 321 droplets and (b,e,f) 643 droplets of silver nanoparticle ink and coated with a layer of acrylate ink. (a,b) show an overview of the protruding 3D electrodes, whilst (c–f) display zoomed images of individual pillars. All images were taken using an accelerating voltage of 15 kV and a substrate tilted at 45°. Different magnifications were used to image the structures: (a,b) 40×, (c,d) 800×, and (e,f) 500×. All pictures except (a,b) show a scale bar of 40 μm, whilst (a,b) exhibit a scale bar of 500 μm, Figure S5: (a) Optical image of the microelectrode array after Pt galvanization in H2PtCl6. (b) Chronoamperometric traces of the individual Ag electrodes during Pt electrodeposition. The scale bar in (a) is 1 mm, Figure S6: (a) Cyclic voltammogram of a Pt-Ag 3D electrode in PBS over 5 cycles. b)-d) tilt-corrected SEM pictures of the 3D electrode after cyclic voltammetry. A magnification of (b) 180×, (c) 1000×, and (d) 5000× were used. All images used an accelerating voltage of 15 kV and a substrate tilted at 45°. (b,c) show a scale bar of 30 μm and (d) 5 μm, Figure S7: Amplifier current noise given for (a) 2D and (b) 3D electrodes in phosphate-buffered saline. In a) the route mean square (RMS) noise was measured for 3 (n = 108), 8 (n = 124), 12 (n = 149), 24 (n = 161), and 150 μm (n = 9) diameter 2D electrodes. In (b) the RMS noise for printed and galvanized 3D electrodes with 128 (n = 7), 192 (n = 15), 321 (n = 7), 643 (n = 12), 964 (n = 5), and 1606 (n = 9) droplets was recorded. The linear fit shown in plot b) has a slope of 130 fA droplet–1, Figure S8: Chip fabrication of functional 3D MEAs viewed from the side (a,c,e,g,i) and the top (b,d,f,h,j). (a,b) PEN substrate, (c,d) printed and thermally sintered Ag feedlines and 3D electrodes, (e,f) Au electrodeposition, (g,h) printed insulation layer for the feedlines, and finally (i,j) Pt electrodeposition.
