# Supplementary material for: Inkjet-Printed and Electroplated 3D Electrodes for Recording Extracellular Signals in Cell Culture
Source: Sensors (Basel). 2021 Jun 9;21(12):3981. doi: 10.3390/s21123981 (PMC8229631; doi:10.3390/s21123981)
Supplement: Supplementary file 1 [file sensors-21-03981-s001.zip › sensors-1243820-supplementary.pdf]

sensors

Supporting Information

Inkjet-printed and electroplated 3D electrodes for recording extracellular signals in cell culture

Leroy Grob[1], Philipp Rinklin[1], Sabine Zips[1], Dirk Mayer[2], Sabrina Weidlich[2], Korkut Terkan[1], Lennart J. K. Weiß[1], Nouran Adly[1], Andreas Offenhäusser[2], and Bernhard Wolfrum[1]*

1 Neuroelectronics, Department of Electrical and Computer Engineering, MSB, MSRM, Technical University of Munich, Boltzmannstraße 11, 85748 Garching, Germany;
2 Institute of Biological Information Processing (IBI-3), Forschungszentrum Jülich GmbH, 52425 Jülich, Germany;
* Correspondence: bernhard.wolfrum@tum.de;

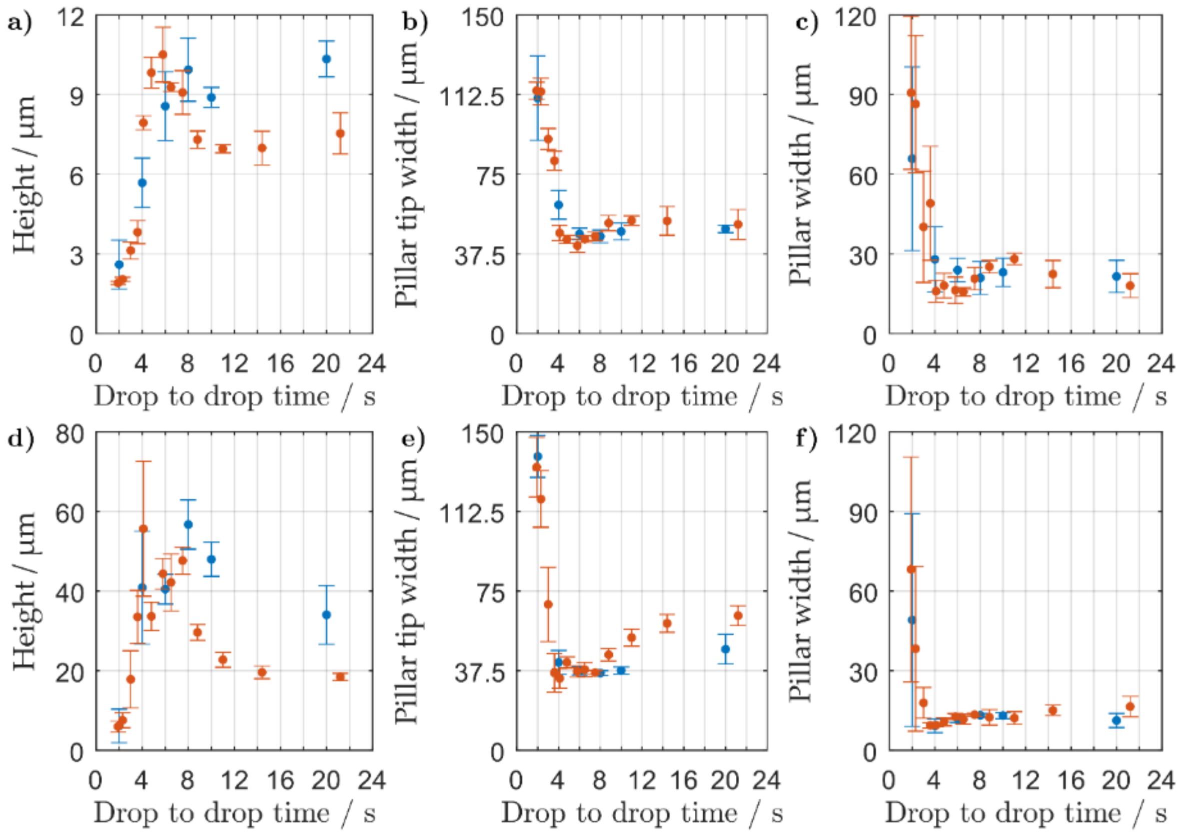

Figure S1. Profilometric data of unsintered pillars printed at different drop-to-drop time intervals with a nozzle plate and sample stage held at 55 and 60 °C, respectively. Stationary inkjet printing (blue) and continuous inkjet printing (red) methods are shown for 3D pillars printed with a)-c) 50 and d)-f) 200 droplets of AgNP ink. a), d) show the height, b), e) the pillar tip width, and c), f) the pillar width. 8-9 pillars were used to calculate the average and the standard deviation shown for blue and red.

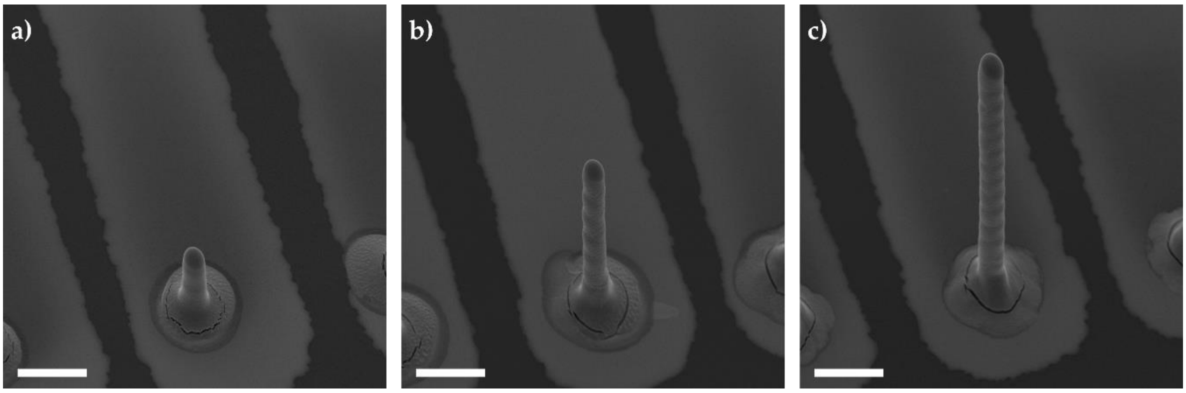

Figure S2. Tilt-corrected scanning electron microscope images of printed and sintered 3D microelectrodes with a) 321 droplets, b) 643 droplets, and c) 964 droplets of AgNP ink. All 3 images were taken using an accelerating voltage of 15 kV, with a magnification of 180×, and a substrate tilted at 45°. The scale bars shown in all images correspond to a length of 100 μm.

Au electrodeposition on Ag 3D MEAs

A constant (CED) or pulsed (PED) voltage waveform was applied to the 3D microelectrodes. Both techniques used the same effective Au deposition time and the results were later compared. For the CED protocol, a constant potential of –1.15 V vs Ag/AgCl (3 M NaCl) was applied for 0, 25, 50, 100, 150, 200, 250, and 300 s. In comparison, the pulsed technique applied a reduction potential of –1.15 V for 20 ms, followed by 0.4 V for 70 ms, and then finally 0 V for 10 ms (all potentials referenced to a 3 M NaCl Ag/AgCl electrode). In total 0, 1250, 2500, 5000, 7500, 10 000, 12 500, and 15 000 pulses were applied. On first inspection, the top-side of the electrodes shown in Figure S3a) showed a significant optical change in color from silver (electrode 1) to golden brown (electrode 8) with increasing deposition interval. The bottom-side (Figure S3b) was still silver in color, with traces of gold mostly around the edges of the feedlines. This would suggest the majority of gold was deposited on the top-side of the printed sensor. Interestingly, electrodes which were galvanized with a deposition interval of 100 s onwards, displayed darker brown features. The brown color is typically associated with an inhomogeneous layering of gold, resulting in a micro- or nano-scopic surface roughness [1]. In comparison to the CED protocol, longer deposition times for the PED protocol did not result in much darkened features (compare Figure S3a and c). On the underside of structures (see Figure S3d) a noticeable layer of Au was also plated within the silver structure. This would indicate a probable reduction in the structural porosity of the structure which has similarly been mentioned in literature [2].

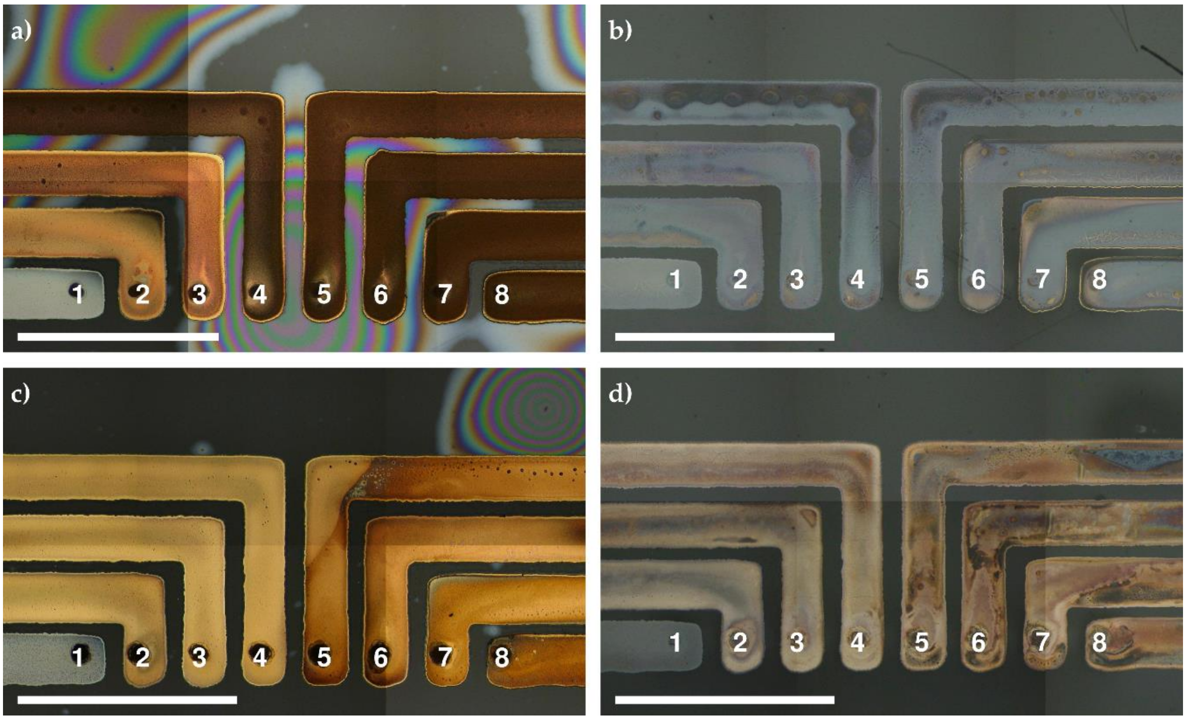

Figure S3. Optical images showing a), c) top, and b), d) underside of Au electroplated silver 3D MEAs. a)-b) were electroplated using a constant potential and c)-d) using a pulsed potential. The effective deposition time of Au was increased from left to right 0, 25, 50, 100, 150, 250, 250, and 300 s (1–8, respectively). The scale bar shown in each picture corresponds to a length of 1 mm.

Analysis of the passivation layer

The thickness of the acrylate insulation layer was measured as the difference in pillar height before and after the passivation step. The height of each 3D electrode was measured using a 3D laser scanning confocal microscope (VK-X250, Keyence, Osaka, Japan) with a 50× objective (50×/0.95 CF Plan Apo OFN25, Nikon, Japan). After curing the passivation, the same pillars were sputtered with Au and viewed under a scanning electron microscope (see section 2.4 for details). Images of the insulated 3D electrodes are shown in Figure S4. The difference in pillar height was measured over 16 pillars where half were printed with 321 and the other half were measured with 643 droplets of ink. The thickness of the deposited acrylate layer was calculated to be 42.2±5.4 µm. Variations in the protruding 3D electrodes are shown in particular in Figure S4b). This is due to the printed Ag structures and not the layer of acrylate ink. The variation in pillar height was most likely due to misaligned droplets, which appear as round patches on the midsection of both 3D electrodes shown in Figure S4e) and f).

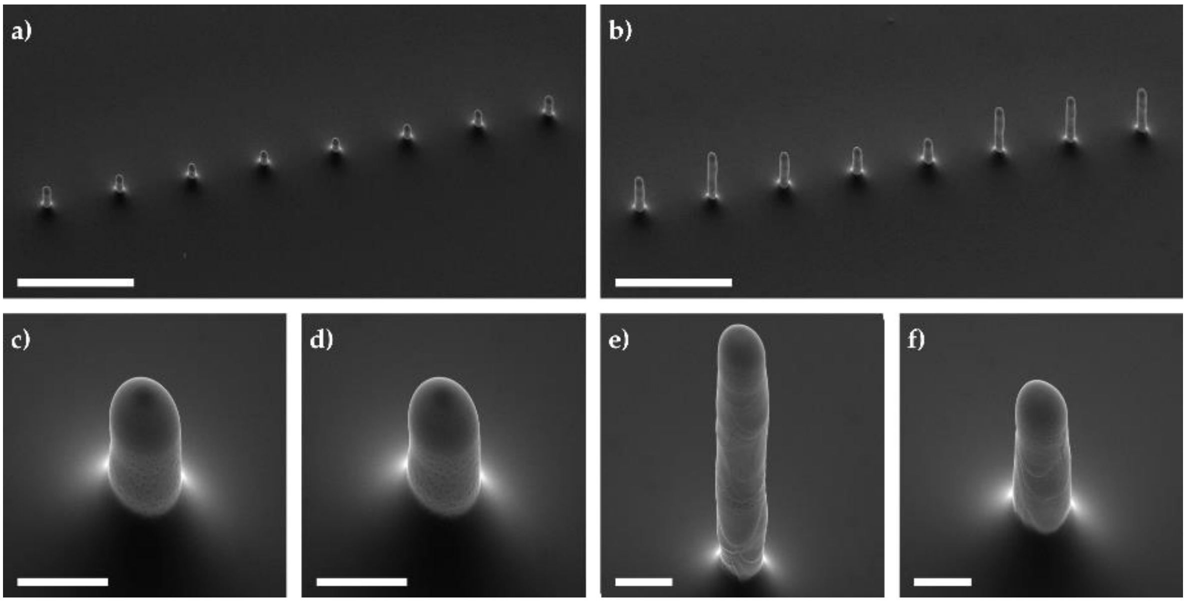

Figure S4. Tilt-corrected scanning electron microscope images of thermally sintered pillars printed with a), c)-d) 321 droplets and b), e)-f) 643 droplets of silver nanoparticle ink and coated with a layer of acrylate ink. a) and b) show an overview of the protruding 3D electrodes, whilst c)-f) display zoomed images of individual pillars. All images were taken using an accelerating voltage of 15 kV and a substrate tilted at 45°. Different magnifications were used to image the structures: a)- b) 40×, c)-d) 800×, and e)-f) 500×. All pictures except a) and b) show a scale bar of 40 µm, whilst a) and b) exhibit a scale bar of 500 µm.

Pt electrodeposition on Ag 3D MEAs

Hexachloroplatanic acid (H_2PtCl_6) is one of the most commonly used platinum based baths [3] due to its high cathode current densities (2.5-3.5 Adm^{-2} at 45-90 °C), allowing a faster mass transport of Pt^{+4} ions to the working electrode in comparison to more basic baths [4]. Due to the bath's possible co-evolution of hydrogen low reduction potentials were applied over 2 min to sintered Ag feedlines ranging from –200mV to –50mV as shown in Figure S5. All Ag structures showed either delamination or cracks resulting in high ohmic values, which are not practical for microelectrode array fabrication. From the chronoamperometric traces, the individual current responses shown in Figure S5b) show a tendency for the Pt deposition to saturate or even abruptly stop as was the case for –160, –170, and –180mV. From literature the mechanisms involved between nanoparticles and plastic films are best described by the general particle adhesion model which is based on van der Waals forces [5]. These forces are weak in comparison to ionic or covalent bonds, which would explain why the acidity of the bath along with the applied reduction potentials were sufficient in damaging the sintered Ag feedlines.

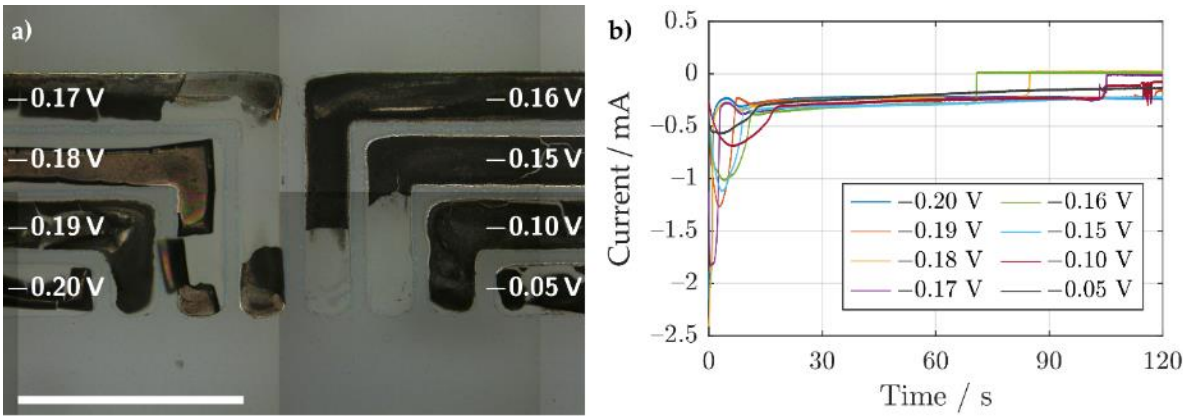

Figure S5. a) Optical image of the microelectrode array after Pt galvanization in H_2PtCl_6. b) Chronoamperometric traces of the individual Ag electrodes during Pt electrodeposition. The scale bar in a) is 1 mm.

Pt electrodeposition on an insulated Ag 3D electrode

In order to protect the feedline structures from the acidic Pt bath, the acrylate passivation was firstly printed, allowing only the 3D Ag electrode to emerge. A pulsed electrodepostion (PED) protocol was used encompassing 12000 pulses, where each pulse comprises a reduction interval for 20 ms held at –0.2 V vs Ag/AgCl (3M NaCl), followed by 0 V vs EOC (electrode's open circuit potential) for 80 ms. This gives an effective reduction interval of 240 s. After the electrodeposition, the electrode was immersed in PBS and swept from –0.4 to 0.8 V for 5 cycles resulting in the cyclic voltammogram in Figure S6a). In comparison to the pulsed electrodeposition of Au, the Pt coated electrode displayed larger distinct oxidation and reduction peaks of Ag (see Figure S6a). This would suggest that Pt was not properly deposited onto the structure, most likely due to the continuous etching of exposed Ag in the acidic electrolyte. After testing the electrochemical stability of the Pt-Ag electrode in electrolyte, the same pillar was viewed under the SEM (see Figure S6b-d). Upon first inspection, the 3D electrode (Figure S6b) looks intact as opposed to the finer feedline structures which were destroyed (Figure S5a). Upon magnification of the electrode's tip depicted in Figure S5) and d), a porous cluster of material can be seen. The material could either be electrodeposited Pt, AgCl formation due to the cyclic sweep in PBS, or a combination of both. Regardless, the electrodeposition of Pt as a single diffusion barrier to Ag is challenging with H2PtCl6.

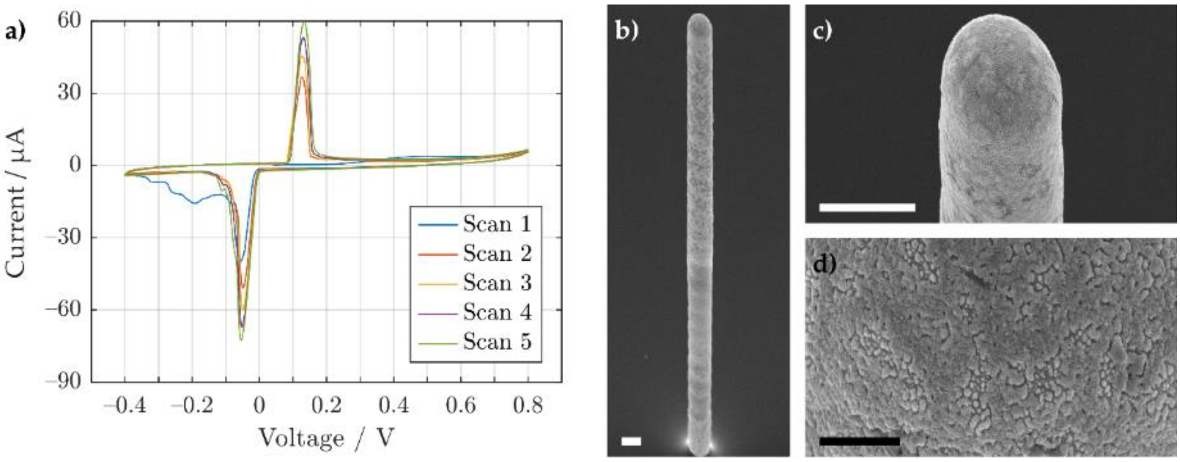

Figure S6. a) Cyclic voltammogram of a Pt-Ag 3D electrode in PBS over 5 cycles. b)-d) tilt-corrected SEM pictures of the 3D electrode after cyclic voltammetry. A magnification of b) 180×, c) 1000×, and d) 5000× were used. All images used an accelerating voltage of 15 kV and a substrate tilted at 45°. b) and c) show a scale bar of 30 µm and d) 5 µm.

Amplifier noise characterization

The noise induced by the electrode–electrolyte interface was characterized using the in-house current amplifier (see section 2.7) and the results are shown in Figure S7. The thermal noise of the amplifier system was firstly measured in an open circuit configuration (i.e. no electrolyte and no inserted reference electrode) and gave a root mean square (RMS) noise of 0.56±0.05 pA (peak-to-peak noise of 5.1±0.5 pA) at a bandwidth of 3.4 kHz. Microfabricated electrodes with a diameter ranging from 3–150 μm were immersed in phosphate-buffered saline and the noise was measured in open circuit (i.e. no inserted reference electrode) shown in Figure S7a). Similarly, printed and galvanized 3D electrodes (fabrication process shown in Figure S8) with varying droplet numbers ranging from 128–1606 droplet were also measured as shown in Figure S7b). In Figure S7a) and b) there is a clear relationship between the electrodes' size and the corresponding noise. With increasing surface area, a larger double-layer capacitance (C_{dl}) is formed which increases the current noise density ($\tilde{i}_n$ with units $A/\sqrt{Hz}$) as described in [6]:

$$\tilde{i}_n = \sqrt{\left(2\pi f \tilde{\varphi}_n C_{dl}\right)^2 + \frac{4k_B T}{R_{FB}}}, \quad (2)$$

where f is the bandwidth of the amplifier, $\tilde{\varphi}_n$ is the overall potential noise of the amplifier ($V/\sqrt{Hz}$), k_B is Boltzmann's constant, T is the temperature and R_{FB} is the feedback resistor of the amplifier. In Figure S7b) the variation in the electrodes' noise between 128–321 droplets is marginal, which can be explained by similarities in the 3D electrodes structure (view Figure 8). These electrodes exhibit a semi-spherical structure, as opposed to electrodes printed with 643 droplets or higher that have a clear pillar structure.

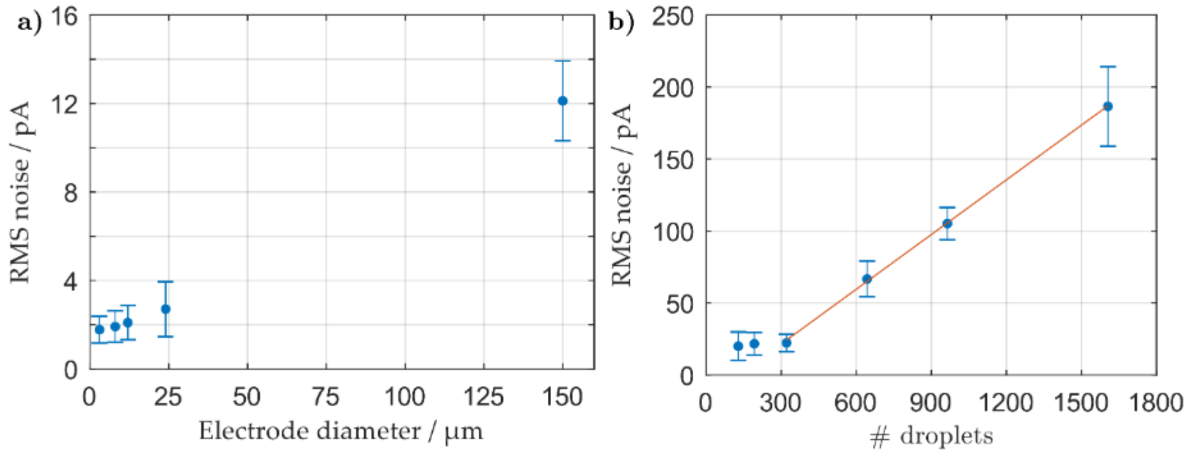

Figure S7. Amplifier current noise given for a) 2D and b) 3D electrodes in phosphate-buffered saline. In a) the route mean square (RMS) noise was measured for 3 (n=108), 8 (n=124), 12 (n=149), 24 (n=161), and 150 μm (n=9) diameter 2D electrodes. In b) the RMS noise for printed and galvanized 3D electrodes with 128 (n=7), 192 (n=15), 321 (n=7), 643 (n=12), 964 (n=5), and 1606 (n=9) droplets was recorded. The linear fit shown in plot b) has a slope of 130 fA droplet^{-1}.

3D electrode fabrication

In the first step of the fabrication process, the substrate was cut to fit the printer's sample stage (shown in Figure A1a and f). Thereafter, the feedlines and 3D electrodes are printed and later thermally sintered at 150 °C for 2 h (illustrated in Figure A1a and f). A glass ring was glued with polydimethylsiloxane (PDMS) to confine the electrolyte for electroplating. Au was electrodeposited onto the 3D MEAs (see Figure A1c and h) using a 3-electrode setup. The printed structures were configured as the working electrode, a larger platinum mesh as the counter electrode and a Ag/AgCl electrode (3M NaCl, RE-6, BASi West Lafayette, USA) was used as the reference. A pulsed waveform was applied to the working electrode with a reduction potential of –1.15 V for 20 ms, followed by 0.4 V for 70 ms, and then finally 0 V for 10 ms (all potentials vs Ag/AgCl reference electrode). This pulse was repeated 10000× which equates to an effective deposition interval of 200 s. The glass ring was carefully removed and an ultra violet curable acrylate ink was printed in between the 3D electrodes to insulate the feedline structures (as shown in Figure A1d and i). Another clean glass ring was glued with PDMS to confine the hexachloroplatinic acid. Pt was electrodeposited onto the 3D MEAs (see Figure A1e and j) using a 3-electrode configuration. A pulsed waveform was applied with a reduction potential of –0.2 V vs Ag/AgCl for 20 ms, followed by 0 V vs EOC (open circuit potential) for 80 ms. This pulse was repeated 24000× which equates to an effective Pt deposition interval of 480 s.

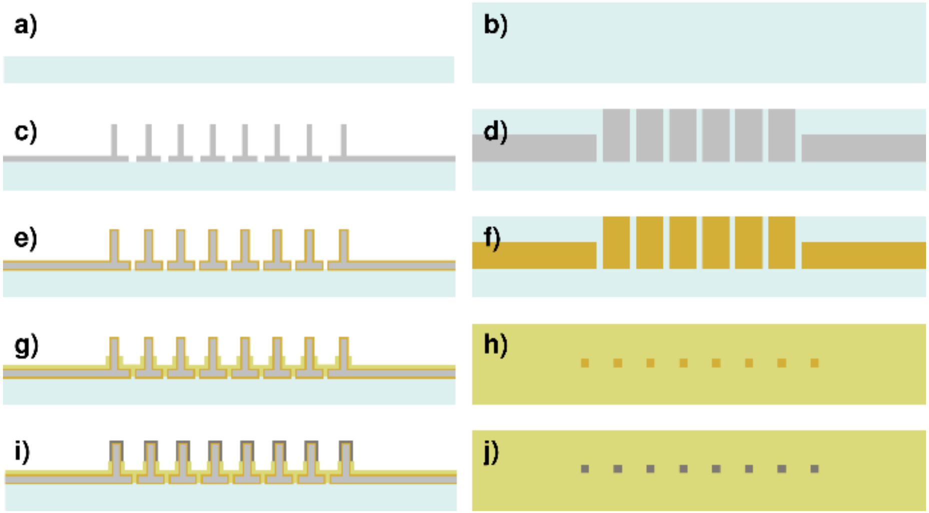

Figure S8: Chip fabrication of functional 3D MEAs viewed from the side (a, c, e, g, and i) and the top (b, d, f, h, and j). a)-b) PEN substrate, c)-d) printed and thermally sintered Ag feedlines and 3D electrodes, e)-f) Au electrodeposition, g)-h) printed insulation layer for the feedlines, and finally i)- j) Pt electrodeposition.

References

1. Brüggemann, D.; Wolfrum, B.; Maybeck, V.; Mourzina, Y.; Jansen, M.; Offenhäusser, A. Nanostructured Gold Microelectrodes for Extracellular Recording from Electrogenic Cells. *Nanotechnology* **2011**, *22*, 265104, doi:10.1088/0957-4484/22/26/265104.
2. Devaraj, G.; Guruviah, S.; Seshadri, S.K. Pulse Plating. *Materials Chemistry and Physics* **1990**, *25*, 439–461, doi:10.1016/0254-0584(90)90111-M.
3. Yasin, H.M.; Denuault, G.; Pletcher, D. Studies of the Electrodeposition of Platinum Metal from a Hexachloroplatinic Acid Bath. *Journal of Electroanalytical Chemistry* **2009**, *633*, 327–332, doi:10.1016/j.jelechem.2009.06.020.
4. Rao, C.R.K.; Trivedi, D.C. Chemical and Electrochemical Depositions of Platinum Group Metals and Their Applications. *Coordination Chemistry Reviews* **2005**, *249*, 613–631, doi:10.1016/j.ccr.2004.08.015.
5. Joo, S.; Baldwin, D.F. Adhesion Mechanisms of Nanoparticle Silver to Substrate Materials: Identification. *Nanotechnology* **2009**, *21*, 055204, doi:10.1088/0957-4484/21/5/055204.
6. Rost, M.J. High-Speed Electrochemical STM. In *Encyclopedia of Interfacial Chemistry - 1st Edition*; Elsevier: 13, 2018; Vol. 1, pp. 180–198.
